# Toxicological and biochemical analyses demonstrate no toxic effect of Cry1C and Cry2A to *Folsomia candida*

**DOI:** 10.1038/srep15619

**Published:** 2015-10-23

**Authors:** Yan Yang, Xiuping Chen, Lisheng Cheng, Fengqin Cao, Jörg Romeis, Yunhe Li, Yufa Peng

**Affiliations:** 1 State Key Laboratory for Biology of Plant Diseases and Insect Pests, Institute of Plant Protection, Chinese Academy of Agricultural Sciences, Beijing 100193, China; 2 College of Environment and Plant Protection, Hainan University, Haikou 570228, China; 3 Qiongtai Teachers College, Haikou 571127, China; 4 Agroscope, Institute for Sustainability Sciences ISS, 8046 Zurich, Switzerland

## Abstract

Collembolans are common soil arthropods that may be exposed to insecticidal proteins produced in genetically engineered (GE) plants by ingestion of crop residues or root exudates. In the present study, a dietary exposure assay was validated and used to assess the lethal and sublethal effects of two *Bacillus thuringiensis* (*Bt*) insecticidal proteins, Cry1C and Cry2A, on *Folsomia candida*. Using the insecticidal compounds potassium arsenate (PA), protease inhibitor (E-64), and *Galanthus nivalis* agglutinin (GNA) mixed into Baker’s yeast, we show that the assay used can detect adverse effects on *F. candida*. Survival and development were significantly reduced when *F. candida* was fed a diet containing PA, E-64, and GNA at 9, 75, and 100 μg/g diet, respectively, but not when fed a diet containing 300 μg/g Cry1C or 600 μg/g Cry2A. The activities of test antioxidant-, detoxification-, and digestion-related enzymes in *F. candida* were unaltered by a diet containing 300 μg/g Cry1C or 600 μg/g Cry2A, but were significantly increased by a diet containing 75 μg/g E-64. The results confirm that Cry1C and Cry2A are not toxic to *F. candida* at concentrations that are much higher than those encountered under field conditions.

Since the appearance of genetically engineered (GE) crops, their potential to cause adverse effects on the environment has drawn much attention. This concern has triggered a regulation requiring that novel GE crop varieties are subjected to rigorous environmental risk assessment before commercialization. One of the risks associated with the planting of GE crops is their potential to harm valued non-target organisms. This is especially relevant for insect-resistant genetically engineered (IRGE) plants including plants expressing *cry* genes derived from various subspecies of *Bacillus thuringiensis* (*Bt*).

When *Bt* crops are grown, the Cry proteins produced by these plants can enter the soil system as a consequence of rhizodeposition before harvest or plant residue decomposition after harvest^1^,^2^,^3^. For example, Li *et al.* monitored the degradation of Cry1Ac in rice stalks after harvest and found that Cry1Ac protein concentration decreased rapidly to 50% during the first month but then more slowly so that the residue rice stalks still contained 21% of the original Cry1Ac concentration after 7 months^2^. A number of studies have indicated that *Bt* proteins released from plant residues or secreted from the roots can be readily adsorbed and bound to soil particles and thus persist in soil^4^,^5^ and maintain their insecticidal activity^5^. Thus, soil organisms have the potential to be exposed to plant-produced *Bt* proteins, therefore assessing the impact of *Bt* proteins or *Bt* plant tissue is part of the non-target risk assessment of GE crops^6^,^7^.

Collembolans, such as *Folsomia candida* (Isotomidae), are commonly found in plant rhizospheres, including those of IRGE rice plants, where they can be exposed to transgene-derived proteins exuded from roots. Furthermore, collembolans play a role in the decomposition of organic matter and may therefore be exposed to transgene-derived proteins that remain in crop residues. Because of their contribution to the decomposition of plant litter and in the formation of soil microstructure, and because they are relatively sensitive to soil quality, collembolans are recognized as suitable indicator species of soil quality and health^8^,^9^. For example, collembolans have been used to assess the quality of polluted soils and of forest and agricultural soil as bio-indicators^10^,^11^,^12^.

The common soil collembolan *F. candida* can be easily maintained in the laboratory^13^,^14^ and is often used to assess the effect of chemical insecticides and insecticidal proteins as part of regulatory risk assessments^7^. Thus, the effects of GE plants on *F. candida* have been assessed under controlled laboratory conditions in many studies^15^,^16^,^17^,^18^. While most previous studies have used GE plant tissue or artificial soil treated with plant tissue in order to expose the test insects to the insecticidal proteins, in the current study we used baker’s yeast to expose *F. candida* to known concentrations of purified Cry proteins. Such dietary exposure assays (often referred to as Tier-1 assays) are regarded as more conservative (i.e., more likely to detect toxic effects) than assays in which test species are exposed to insecticidal compounds by feeding on GE plant tissue or are exposed in other ways^19^,^20^,^21^.

In the present study, we validate a dietary exposure assay for evaluating the potential effects of oral insecticidal compounds on *F. candida*. We then use the assay to assess the potential effects of Cry1C or Cry2A on *F. candida* survival and development because relatively little is known regarding the sensitivity of species belonging to the Collembola compared to other arthropod taxa^22^. In addition, the activity of antioxidant-, detoxification-, and digestion-related enzymes in *F. candida* was measured after the collembolan was fed Cry1C or Cry2A protein because changes in enzyme activities would indicate potential sublethal effects of the *Bt* proteins.

## Results

### Response of *F. candida* to PA, E-64, or GNA

**Figure 1:**
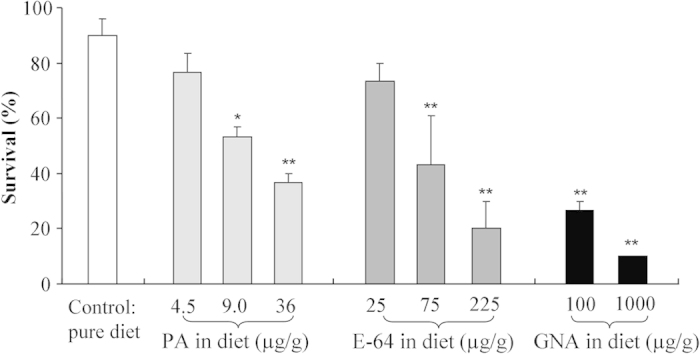
Survival of *Folsomia candida* when fed artificial diets containing different concentrations of PA, E-64, or GNA for 28 d. Values are means + SE, n = 3. Asterisks indicate significant differences between the toxin treatment and the control (*P < 0.05, **P < 0.01).

Potassium arsenate (PA), the protease inhibitor E-64, and *Galanthus nivalis* agglutinin (GNA) were used to validate the test system because their toxicity to *F. candida* were established in a preliminary experiment. In the pure diet control treatment, the survival rate of *F. candida* after 28 days of feeding was >85%. With increasing concentrations of PA, E-64, or GNA in the diet, the survival rate of *F. candida* steadily declined (Fig. 1). Relative to survival on the control diet, survival was significantly reduced on diets containing PA at ≥9.0 μg/g FW diet, E-64 at ≥75 μg/g FW diet, or GNA at ≥100 μg/g FW diet (Dunnett’s test: all *P* < 0.03) (Fig. 1).

Independent from the treatment, *F. candida* increased in size during the duration of the experiment (till day 28) (Table 1). However, growth was significantly reduced in the PA, E-64and GNA treatments when compared to the untreated control. When compared to the control, the body length of *F. candida* was significantly reduced by day 7 at the highest concentration of PA (36 μg/g FW diet), while at day 14, 21, and 28, significant reductions were caused by PA at 9.0 μg/g FW diet (*P* < 0.05). The head width of *F. candida* was significantly reduced by PA at ≥9.0 μg/g FW diet from day 7 onwards (all P < 0.01). For the diets containing E-64, significant reductions in body length were recorded with 75 μg/g FW diet from day 21 onwards and from day 7 onwards at the highest concentration of 225 μg/g FW diet (P < 0.05). For diets containing GNA, 100 μg/g FW diet significantly reduced both body length and head width by day 7 (Table 1).

### Toxicity of Cry proteins to *F. candida*

#### Effects on life-table parameters

**Figure 2:**
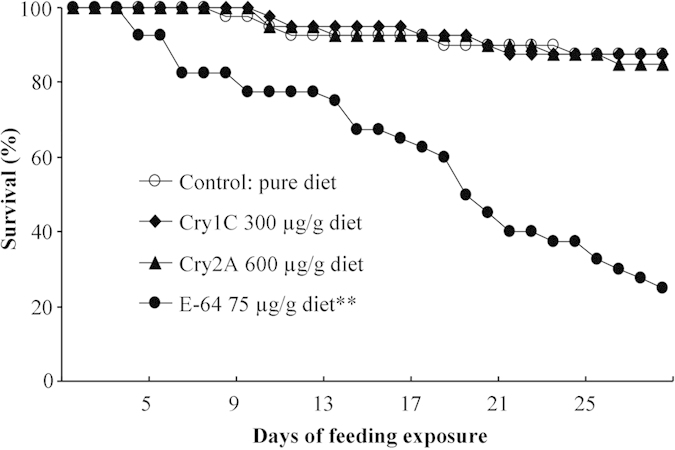
Survival of *Folsomia candida* when fed a pure artificial diet (negative control) or diets containing Cry1C, Cry2A, or E-64 (positive control). Asterisks indicate a significant difference between the treatment and the negative control (*P* < 0.01) (n = 40).

**Figure 3:**
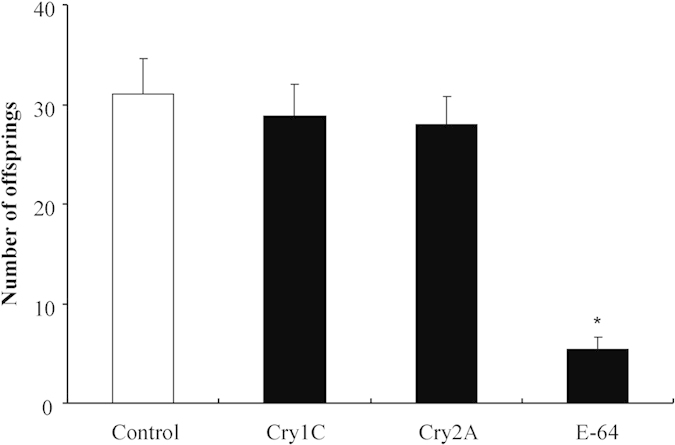
Numbers of offspring produced per *Folsomia candida* when fed a pure artificial diet (negative control) or diets containing Cry1C, Cry2A, or E-64 (positive control). Values are means + SE, n = 40. Asterisks indicate a significant difference between the treatment and the negative control (*P* < 0.05).

The survival rates of *F. candida* were >85% when fed a pure diet or a diet containing Cry1C or Cry2A at 300 or 600 μg/g diet, respectively, and pair-wise comparisons revealed no significant difference between survival with Cry1C or Cry2A and the untreated control (χ^2^ = 0.00, *P* = 0.984 for Cry1C; χ^2^ = 0.08, *P* = 0.774 for Cry2A) (Fig. 2). In contrast, *F. candida* survival was significantly reduced by feeding on a diet containing E-64 at 75 μg/g diet (χ^2^ = 31.526, *P* < 0.001). Similarly, *F. candida* body length and head width were not affected by Cry1C and Cry2A (Dunnett’s test; all *P* > 0.1) but were significantly reduced by E-64 feeding when compared to the control (*P* < 0.05) (Table 2). In addition, the mean number of offspring produced per insect during the 28 days of feeding was not significantly affected by Cry1C or Cry2A (Dunnett’s test; *P* > 0.05) (Fig. 3) but was significantly reduced by E-64 (*P* < 0.001).

#### Uptake of Cry proteins by F. candida

ELISA measurements showed that all *F. candida* fed with Cry protein-incorporated diets contained Cry proteins. The mean concentrations (±SE, n = 3) of Cry1C detected in *F. candida* on days 14, 21, and 28 were 12.0 ± 2.0, 12 ± 1.0, and 28.0 ± 2.0 ng/g dry weight (DW), respectively, and changed significantly over time (RM-ANOVA, *F* = 35.8, df = 2, *P* = 0.003). The mean concentrations of Cry2A detected in *F. candida* on days 14, 21, and 28 were 19.0 ± 5.0, 9.0 ± 1.0, and 7.0 ± 1.0 μg/g DW, respectively. The Cry2A concentration decreased over time but this was not significant (*F* = 8.7, df = 2, *P* = 0.064). No Cry protein was detected in *F. candida* fed on untreated control diet.

#### Stability and bioactivity of Cry proteins

Based on ELISA, the quantity of the two Cry proteins detected in the yeast powder diet supplemented with the proteins ranged from 66 to 88% of the nominal concentrations. The mean (±SE) concentration detected in the freshly prepared diet was 214.13 ± 10.32 μg/g FW for Cry1C (300 μg/g had been added to the diet) and 514.70 ± 9.66 μg/g FW for Cry2A (600 μg/g had been added to the diet). After 2 days of feeding exposure, the mean Cry protein concentration in the diet had declined by 10.9 to 17.0%. This decline was significant for Cry2A (442.9 ± 13.9 μg/g; Student’s *t*-test; *t* = 4.25, *df* = 4, *P* = 0.01) but not for Cry1C (186.9 ± 6.80 μg/g; *t* = 2.21, *df* = 4, *P* = 0.09).

Sensitive-insect bioassays were conducted to measure the bioactivity of Cry proteins in the yeast powder diet. The results showed that the mortality of *Chilo suppressalis* larvae was 0% when the larvae were fed a diet that contained the extract from the untreated yeast diet for 7 days. When *C. suppressalis* larvae were fed diets containing the extracts from freshly prepared Cry-containing yeast diets, the mortalities were 96.7% and 73.3% for Cry1C and Cry2A treatments, respectively; these values were not significantly different from those obtained with extracts from Cry-containing diets that had been exposed to *F. candida* for 2 days (Chi-square test; U = 2.965, df = 1, *P* = 0.195 for Cry1C; U = 0.32, df = 1, *P* = 0.779 for Cry2A).

### Enzyme activities in *F. candida*

**Figure 4:**
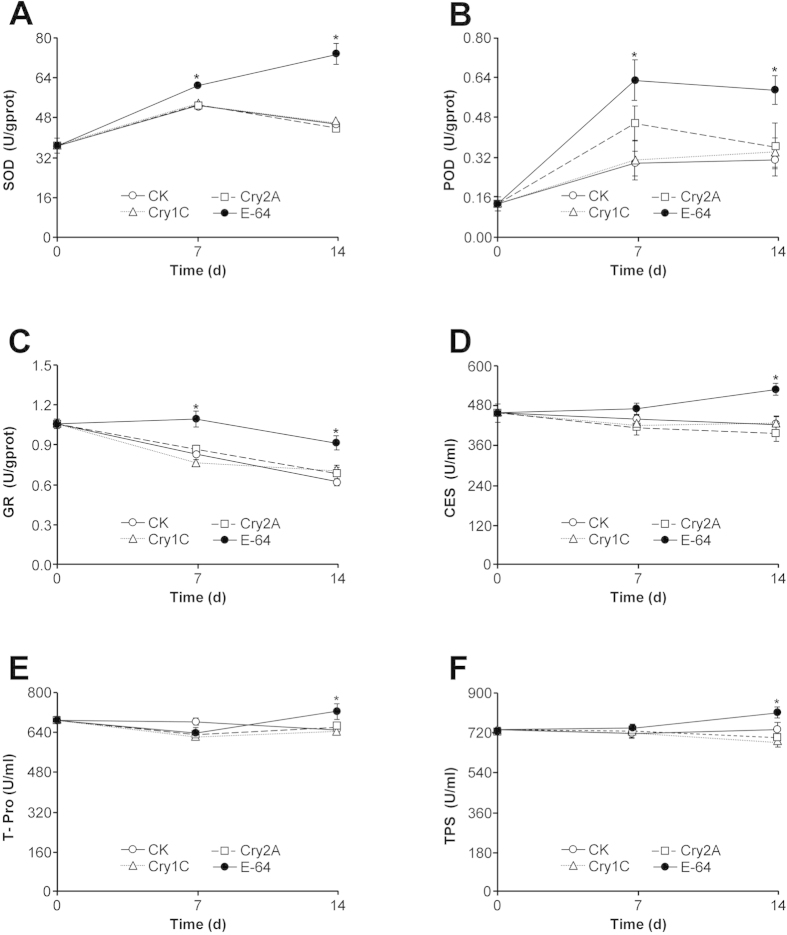
Enzyme activities (U) in *Folsomia candida* when the collembolan was fed a pure artificial diet (negative control) or diets containing Cry1C, Cry2A, or E-64 (positive control) for 0, 7, or 14 days. The following enzymes were analysed: (**A**) superoxide dismutase (SOD); (**B**) peroxidase (POD); (**C**) glutathione reductase (GR); (**D**)carboxylesterase (CES); (**E**) total proteases (T-Pro); (**F**) tryptase (TPS). Values are means + SE, n = 10. Asterisks indicate a significant difference between the treatment and the negative control (*P* < 0.05).

We measured the activity of six enzymes in *F. candida* and none were shown to be significantly different in individuals fed diets containing Cry1C or Cry2A compared with individuals fed untreated diet (Dunett’s test; *P* > 0.05) (Fig. 4). In contrast, the activity of all six enzymes was significantly increased in *F. candida* when the diet contained E-64. For SOD, POD, and GR, the increase was significant on day 7 and day 14 (SOD: *P* < 0.001 on day 7 and day 14; POD: P = 0.006 on day 7 and P = 0.028 on day 14; GR: *P* < 0.001 on day 7 and day 14) (Fig. 4A–C). For CES, T-Pro or TPS, the activity was not significantly increased on day 7 (*P* > 0.05) but was significantly increased on day 14 (CES: *P* = 0.006; T-Pro: *P* = 0.029; TPS: *P* = 0.02) (Fig. 4D–F).

## Discussion

Evaluation of the potential negative effects of an IRGE crop on non-target arthropods is an important part of the environmental risk assessment that is performed before a GE crop can be commercialized^6^. A typical hypothesis to be tested is that the plant-produced insecticidal compound does not harm the valued non-target species at concentrations present in the field, and a tiered framework has been recommended and widely accepted for such assessments^6^,^23^,^24^. The assessments typically begin with laboratory experiments, namely Tier-1 assays, whose main objective is to test the potential hazard of insecticidal proteins such as *Bt* proteins produced by IRGE plants on the surrogate species under controlled, worst-case exposure conditions^20^,^21^,^24^.

Ideally, a suitable artificial diet is available and can serve as a carrier for delivering the insecticidal proteins to the test organism^20^,^21^. In the current study, yeast powder was used as a diet for *F. candida.* When *F. candida* was fed with this diet, its survival was >85% during the 28-day feeding period, which fulfilled the criteria of such assays^20^. The test Cry proteins and the positive control compounds could be easily and uniformly mixed with the yeast powder, indicating that this diet is suitable for delivering test compounds to *F. candida*. The compounds E-64, GNA, and PA generated dose-dependent declines in *F. candida* survival and growth (measured as body length and head width) when compared to untreated control diet. This indicated that the experimental system was capable of detecting dietary effects caused by gut-active insecticidal compounds and that the compounds could serve as positive controls in subsequent bioassays. E-64 was selected as a positive control compound for the subsequent assays because it is less expensive than GNA and because PA may also cause some contact toxicity.

Once the new bioassay was found to be useful for detecting toxicity, the toxicity of purified Cry1C and Cry2A to *F. candida* was evaluated. The two proteins were selected because the transgenic rice line T1C-19 expresses *cry1C* and the transgenic rice line T2A-1 expresses *cry2A*; these lines were recently developed in China and exhibit high resistance to the target lepidopteran pests^25^. Our results indicate that the two Cry proteins have no adverse effects on the fitness of *F. candida*, even though the concentrations used were significantly higher than the highest concentrations of the Cry proteins contained in the tissues of the two *Bt* rice lines^25^. A diet containing E-64 significantly reduced survival, development, and reproduction of *F. candida*. This positive control suggests that the test proteins were actually ingested and again demonstrates that our experimental system was able to detect adverse effects caused by toxic compounds.

That the Cry proteins were ingested by *F. candida* in our assay was confirmed by ELISA. ELISA also indicated that >80% of the Cry proteins were still detectable in the diet after the 2-day feeding exposure. Furthermore, the bioactivity of the Cry proteins in the yeast diet was confirmed by a bioassay with larvae of the *Bt* protein-sensitive *C. suppressalis.* These results demonstrate that *F. candida* larvae were exposed to bioactive Cry1C and Cry2A protein in our feeding study. The results reinforce the conclusion that the species is not sensitive to the two Cry proteins. While Sims & Martin have already reported a lack of toxicity of Cry2A to *F. candida*^26^, we are not aware of any study that has assessed the impact of Cry1C. Similar dietary studies have been conducted for other beneficial arthropods, such as the green lacewing *Chrysoperla sinica* (Neuroptera: Chrysopidae), the ladybird beetle *Propylea japonica* (Coleoptera: Coccinellidae), and the honeybee *Apis mellifera* (Hymenoptera: Apidae); none of these studies reported any effect of feeding on a diet containing Cry1C or Cry2A on insects outside the order of Lepidoptera^27^,^28^,^29^,^30^.

To determine whether the tested Cry proteins might have sublethal effects on *F. candida*, we measured the activities of antioxidant enzymes (SOD and POD), detoxification enzymes (GR and CES), and the proteases (T-Pro and TPS) after the collembolan had fed on a diet containing Cry1C, Cry2A, or E-64 for 7 and 14 days. SOD converts O_2_ to H_2_O_2_ through dismutation, and H_2_O_2_ is subsequently turned into H_2_O by POD; this series of reactions reduces or eliminates the damage that O_2_ can cause to membranes^31^. CES hydrolyzes ester, amide, and carbamate bonds and is important in pesticide and lipid metabolism^32^,^33^,^34^. GR is a flavoprotein that catalyzes the NADPH-dependent reduction of glutathione disulfide (GSSG) to glutathione (GSH); the reaction is essential for the maintenance of glutathione levels. Glutathione has a major role as a reductant in oxidation–reduction processes, serving in detoxification and several other important cellular functions^35^. T-Pro is widely used as an indicator of an insect’s adaptation to food; TPS has been suggested to be a key mediator in insects for resistance evolution to *Bt* insecticidal proteins^36^. The activities of such enzymes have been widely used as indicators for adverse effects caused by stomach poisons in arthropods^17^,^37^,^38^,^39^. The results from our study show that the activities of the test enzymes in *F. candida* were not affected by feeding on Cry1C or Cry2A. The results agree with those reported from previous studies. For example, Yuan *et al*. reported that the activity of antioxidant enzymes including SOD and POD in *F. candida* was not affected when the collembolan was fed yeast mixed with Cry1Ab and Cry1Ac proteins^39^. Bai *et al.* also found that the SOD activity was not significantly altered in *F. candida* after the collembolan had fed on Cry1Ab-containing rice tissue for 35 days^17^. To our knowledge, the current study is the first to measure the activities of the detoxification enzymes GR and CES and the proteases T-Pro and TPS in *F. candida* as a response to Cry protein uptake. Whereas the activities of the test enzymes in the current study were unaffected by the addition of Cry1C or Cry2A to the diet, the activities of all tested enzymes were significantly increased when *F. candida* was fed a diet containing E-64, which was toxic to the collembolan in the previous assays.

Because of their ecological importance as decomposers of plant litter in soil, collembolans in general and *F. candida* in particular have received much attention in the environmental risk assessment of IRGE crops. In most previous studies, *F. candida* was exposed to the Cry proteins by providing them with tissue from transgenic plants or with a mixture of plant tissue and soil. While most of these studies did not detect any adverse effects when *F. candida* fed on *Bt* plant tissue vs. non-*Bt* plant tissue^17^,^18^,^39^,^40^,^41^, negative effects were detected in a few studies. For example, *F. candida* produced significantly fewer fecal pellets when fed *Bt* (Cry1Ab) maize tissue rather than non-*Bt* maize tissue^42^,^43^. In such cases, however, it is not possible to determine whether the negative effects were caused by the Cry proteins or by other differences in the composition of the *Bt* vs. the non-*Bt* plants. In addition, plant material is less suitable than yeast as a food for *F. candida*^44^. By using the dietary exposure assay developed in the current study, we were able to measure the direct toxicity of Cry proteins to *F. candida* without confounding differences in plant tissue composition or other factors. In addition, the concentrations of the test compounds can be adjusted in the new assay, and the test species can be exposed to much higher concentrations of the test compounds than would be encountered in the field under realistic conditions which adds certainty to the conclusion of no effects^6^,^19^,^20^,^21^.

In summary, toxicological and biochemical techniques were used to assess the potential toxicity of Cry1C and Cry2A proteins to *F. candida*, and the results demonstrated that *F. candida* is insensitive to both Cry proteins. The results were consistent with previous reports that purified *Bt* proteins Cry1Ab, Cry1Ac, Cry2A, and Cry3A are not toxic to *F. candida*^26^,^39^,^45^. More importantly, the study describes a dietary exposure system that can be used to assess the direct toxic effects of orally active insecticidal compounds on *F. candida*.

## Materials and Methods

### Test organism

*Folsomia candida* was obtained from the Shanghai Institute for Biological Sciences, Chinese Academy of Sciences. Insects were cultured in Petri dishes (diameter 90 mm; height 10 mm) filled with a solidified mixture of plaster of Paris, activated charcoal, and distilled water at a ratio of 9 : 1 : 10 (w : w : w) and with a height of 3–5 mm (hereafter referred to as plaster-based Petri dishes). The plaster of Paris and activated charcoal base were kept moist by regularly adding distilled water, which resulted in a relative humidity close to 100% in the Petri dishes. Baker’s yeast (AB MAURI, Heben Mauri Foods Co., Ltd., Zhangjiakou, China) was provided as food for the insects and was renewed weekly to reduce fungal and bacterial contamination. The Petri dishes were kept in a dark growth chamber at 20 ± 1 °C. Insects used in the experiments were 10–12 days old.

### Insecticidal compounds

Insecticidal compounds used in this study included lyophilized GNA, PA (KH_2_AsO_4_), protease inhibitor E-64 [(2S,3S)-trans-Epoxysuccinyl-L-leucylamido-3-methylbutane ethyl ester EST], and the *Bt* proteins Cry1C and Cry2A. GNA, PA, and E-64 were purchased from Sigma–Aldrich (St. Louis, MO), and the *Bt* proteins were purchased from Envirotest-China (agent for EnviroLogix Inc., Portland, Maine, USA; www.envirotest-china.com). The protoxins from *Bt* had been expressed as single-gene products in *Escherichia* coli (Cry1C) or in a cured *Bt* strain (Cry2A) at Case Western Reserve University (USA). The protoxin inclusion bodies were then dissolved and trypsinized, and then isolated and purified by ion exchange HPLC followed desalting and lyophilizing the pure fractions. The purity ranged from 94–96% (Marianne P. Carey, Case Western Reserve University, personal communication).

The bioactivity of the Cry proteins was confirmed by sensitive-insect, laboratory bioassays using neonate larvae of *C. suppressalis* that were fed an artificial diet containing a range of Cry protein concentrations for 7 days. The EC_50_ (toxin concentration resulting in 50% weight reduction compared to the control) was estimated to be 21.66 and 1302.60 ng/g for Cry1C and Cry2A, respectively (see Supplementary Information).

### Artificial diet

Development of a robust and reliable dietary exposure assay requires an appropriate artificial diet to deliver the test compounds to the test organisms^21^. Baker’s yeast powder is an excellent food for *F. candida*^44^,^46^ and was used in the current study to deliver the test compounds. To incorporate Cry proteins, GNA, E-64, and PA into yeast powder, the compounds were first dissolved in distilled water at a defined concentration and then mixed with yeast powder at the ratio of 1 : 5 (w : w). The mixture was then lyophilized and ground into powder again. The diet was kept at −20°C until it was fed to *F. candida*.

### Response of *F. candida* to PA, E-64, and GNA

Appropriate positive controls are necessary for a dietary exposure assay^21^. Bioassays were conducted to determine whether the yeast diet is appropriate for testing the toxicity of insecticidal compounds to *F. candida* and to clarify which of three compounds (GNA, PA, or E-64) is the most appropriate for use as the positive control in feeding assays with *F. candida*. Stock solutions of PA, E-64, and GNA were diluted with distilled water and incorporated into yeast powder as described in the previous section to obtain the following toxin concentrations: 4.5, 9, 18, and 36 μg/g fresh weight (FW) of diet for PA; 25, 75, and 225 μg/g FW of diet for E-64; and 100 and 1000 μg/g FW of diet for GNA. These concentrations were selected based on our preliminary experiments and the results from previous studies with other insect species^47^,^48^,^49^,^50^,^51^. Yeast powder treated with pure distilled water served as the negative control.

Adults of *F. candida* were placed in plaster-based Petri dishes and allowed to oviposit for 48 h before being removed. When the eggs hatched after approximately 7 days, the neonates were fed with untreated yeast powder for 11 days before they were used in the experiments. The age of the test organisms has been selected for two reasons: (i) According to the OECD test protocol^52^, it is recommended to use well fed *F. candida* at an age between 9 and 12 days; (ii) *F. candida* at this age have successfully been used to assess the impact of GM plant tissue or Cry proteins^16^,^26^. The insects were randomly selected and 10 individuals were kept in each plaster-based Petri dish, where they were fed with control diet or insecticide-treated diets as described earlier. The diets were replaced every 2 days. Each combination of insecticide and concentration was represented by three replicate, namely 3 Petri dishes with 10 insects per dish. Survival of the insects was recorded twice per day (9:00 am and 9:00 pm), and body length and head width were measured every 7 days. For measurement, pictures of the test organisms were taken by photomicroscope and subsequently body length and width were measured using a staff gauge. All living insects were measured. The experiment was terminated after 28 days. The bioassay was conducted in a climate chamber at 20 ± 1 °C with 70 ± 5% RH and a 12-h light/12-h dark cycle.

### Toxicity of Cry proteins to *F. candida*

#### Effects on life-table parameters

The same experimental procedure that was used to assess the toxicity of PA, E-64, and GNA was used to assess the toxicity of Cry1C and Cry2A to *F. candida*. In contrast to the previous experiment, *F. candida* were individually kept in Petri dishes, and 40 insects were tested (40 replicates) in each treatment. The following four dietary treatments were tested: i) diet containing Cry1C protein; ii) diet containing Cry2A protein; iii) diet containing E-64 (positive control); and iv) diet containing no toxin (negative control). The nominal concentrations of Cry1C, Cry2A, and E-64 proteins in diets were 300, 600, and 75 μg/g FW diet, respectively. The Cry1C and Cry2A protein concentrations used are >10-times greater than the mean concentrations in different tissues of *Bt* rice lines recently developed in China (Cry1C: 0.90-3.65 μg/g FW leaves of T1C-19b rice; Cry2A: 0.76-87μg/g FW leave of T2A-1 rice)^25^. The concentration of E-64 was selected based on the results from the bioassay described earlier in this paper. Diets were prepared 3 days before initiation of the experiment and were stored at −20 °C until used. Diets were renewed every 2 days to prevent the degradation of the test compounds.

Survival of *F. candida* was assessed twice per day (9:00 am and 9:00 pm). The body length and head width were measured every 7 days as described above. The experiment was terminated after 28 days, at which time all *F. candida* in each Petri dish, including larvae and unhatched eggs, were counted.

#### Uptake of Cry protein by F. candida

More than 1000 *F. candida* (10–12 days old) were fed with diet containing no toxin, 300 μg/g Cry1C, or 600 μg/g Cry2A for 28 days as described earlier. Insects were collected after 14, 21, and 28 days. Fifty to 60 individuals were collected for each sample, and three samples were collected at each sampling date resulting in a total of 27 samples. The insect samples were frozen at −80 °C for ELISA analyses (see below).

#### Stability and bioactivity of Cry proteins

The temporal stability and bioactivity of the Cry proteins in the artificial diets were measured in three subsamples (2–3 mg FW of diet per subsample) that were collected from fresh diets taken from the freezer and from diets that had been exposed to *F. candida* for 2 days. The Cry protein concentrations and bioactivities were determined by ELISA and by a ‘‘sensitive-insect’’ bioassay as described in the following sections.

#### ELISA analyses

The Cry protein concentrations in *F. candida* samples and in artificial diet were measured by double-antibody sandwich enzyme-linked immunosorbent assays (DAS-ELISA) using Cry1C and Cry2A detection kits purchased from Enviro-Logix (Portland, Maine, USA). Before the analyses, all insects were washed in phosphate-buffered saline Tween (PBST) to remove any *Bt* toxin from their outer surface. For Cry protein extraction, samples of insects or artificial diets were weighed and mixed with PBST at a ratio of at least 1:10 to 1:100 (mg of sample : μl of buffer) in 1.5-ml centrifuge tubes. The samples were then fully ground by hand using an electric grinding rod. After centrifugation and appropriate dilution of the supernatants, ELISA was performed according to the manufacturer’s instructions. The optical density (OD) values were read with a microplate spectrophotometer (PowerWave XS2, BioTek, USA). The concentrations of Cry1C and Cry2A were calculated by calibrating the OD values to a range of concentrations of standard Cry1C and Cry2A samples.

#### Sensitive-insect bioassay

*Bt*-susceptible *C. suppressalis* larvae were used as sensitive insects to verify the bioactivity of the Cry proteins in the yeast diets before and after the 2-days feeding exposure. The Cry proteins were firstly extracted from the yeast diet samples as described in the ELISA analyses section, and the supernatants were appropriately diluted before being incorporated into the artificial diet for *C. suppressalis*^53^. The *C. suppressalis* diet must be heated during preparation. To avoid the degradation of the Cry proteins during heating, the supernatants were mixed into the diet when the temperature had decreased to less than 60 °C. Once the diet was solid, it was cut into slices and individually placed in Petri dishes (90 mm diameter, 15 mm height). Neonates of *C. suppressalis* were individually transferred to the Petri dishes, which were subsequently sealed with Parafilm. Thirty replicates were tested for each treatment. After 7 days, the mortality of *C. suppressalis* larvae in each treatment was recorded.

### Determination of enzyme activity

*F. candida* larvae (10 to 12 days old) were exposed to 300 μg Cry1C/g diet or 600 Cry2A μg/g diet for 0, 7, and 14 days using the same procedure described earlier. At each sampling date, 200 to 300 *F. candida* were collected and stored at −20 °C before the activities of the following enzymes were quantified: digestion-related enzymes (total protease, T-Pro) and tryptase, TPS); the antioxidant-related enzymes (superoxide dismutase, SOD and peroxidase, POD); and the detoxification-related enzymes (carboxylesterase, CES and glutathione reductase, GR). SOD, POD, and GR activities were measured with ELISA kits from Nanjing Jiancheng Ltd. Co. (Nanjing, China), and TPS, CES, and T-Pro activities were measured with ELISA kits from Beijing Luyuan Byrd Biological Technology Ltd., Co. (Beijing, China).

Insect samples were homogenized at 4 °C in physiological saline solution at a ratio of 1 : 9 (w : v). The homogenates were then centrifuged at 2500–3000 × *g* for 10 min at 4 °C, and the resulting supernatants were used for analysis of the enzyme activities following the manufacturer’s instructions. The optical density (OD) values were read with a microplate spectrophotometer (PowerWave XS2, BioTek, USA). The activities of the enzymes were calculated by calibrating the OD values to a range of concentrations of standards provided with the kits.

### Data analysis

In all bioassays with *F. candida*, statistical comparisons were made between each treatment and the control (pure diet). Data on body length, head width, and the number of offspring were compared by Dunnett’s tests. The offspring data were transformed by log (x) to satisfy the assumptions of parametric analysis (normal distribution of residues and homogeneity of error variances). In the bioassays with PA, E-64, and GNA, the survival rates were analyzed by Dunnett’s tests after the data were transformed by SQRT(x + 1). The effect of Cry protein dietary treatments on *F. candida* survival was analyzed with the Kaplan-Meier procedure and Logrank test.

Cry protein concentrations in *F. candida* collected on different days during the feeding assay were analysed by repeated-measures ANOVA to test for changes in protein uptake over time. In addition, Student’s *t*-tests were used to compare Cry protein concentrations in the fresh diet *vs.* diet exposed to *F. candida* larvae for 2 days. Chi-square tests were used to compare the mortality of the *C. suppressalis* larvae that were fed with artificial diets containing the extracts from: untreated pure yeast diet; yeast diet containing fresh Cry; and yeast diet containing Cry that had been exposed to *F. candida* larvae for 2 days. The enzyme activities were compared between each toxin treatment and the pure diet control using Dunnett’s tests.

All statistical analyses were conducted using the software package SPSS (version 13 for windows, 2004).

## Additional Information

**How to cite this article**: Yang, Y. *et al.* Toxicological and biochemical analyses demonstrate no toxic effect of Cry1C and Cry2A to *Folsomia candida*. *Sci. Rep.* **5**, 15619; doi: 10.1038/srep15619 (2015).

## Supplementary Material

Supplementary Information

## Figures and Tables

**Table 1:** Body length and head width of *Folsomia candida* when fed artificial diets containing different concentrations of PA, E-64, or GNA for 7, 14, 21, or 28 days. Values are means ± SE, n = 3.

Treatment	Body length (mm)				Head width (mm)			
	Day 7	Day 14	Day 21	Day 28	Day 7	Day 14	Day 21	Day 28
Control: pure diet	0.901 ± 0.008	1.169 ± 0.024	1.272 ± 0.054	1.329 ± 0.036	0.231 ± 0.005	0.241 ± 0.005	0.251 ± 0.004	0.272 ± 0.007
PA 4.5 μg/g diet	0.897 ± 0.029	1.108 ± 0.031	1.224 ± 0.025	1.283 ± 0.016	0.229 ± 0.008	0.235 ± 0.004	0.247 ± 0.003	0.265 ± 0.008
PA 9.0 μg/g diet	0.823 ± 0.020	1.029 ± 0.040^*^	1.084 ± 0.017^**^	1.085 ± 0.029^**^	0.196 ± 0.003^**^	0.196 ± 0.005^**^	0.215 ± 0.003^**^	0.221 ± 0.007^**^
PA 36 μg/g diet	0.798 ± 0.015^*^	0.960 ± 0.012^**^	–	–	0.186 ± 0.005^**^	0.197 ± 0.004^**^	–	–
E-64 25 μg/g diet	0.924 ± 0.032	1.135 ± 0.022	1.191 ± 0.012	1.257 ± 0.027	0.228 ± 0.002	0.237 ± 0.008	0.250 ± 0.002	0.261 ± 0.005
E-64 75 μg/g diet	0.832 ± 0.006	1.108 ± 0.023	1.078 ± 0.046^*^	1.156 ± 0.013^**^	0.200 ± 0.003^*^	0.211 ± 0.005^**^	0.214 ± 0.006^**^	0.216 ± 0.009^**^
E-64 225 μg/g diet	0.791 ± 0.005^**^	0.917 ± 0.039^**^	–	–	0.187 ± 0.005^**^	0.206 ± 0.002^**^	–	–
GNA 100 μg/g diet	0.836 ± 0.006^**^	1.074 ± 0.011^*^	1.096 ± 0.023^*^	–	0.194 ± 0.004^**^	0.205 ± 0.006^**^	–	–
GNA 1000 μg/g diet	0.805 ± 0.012^**^	1.015 ± 0.007^**^	1.019 ± 0.038^**^	–	0.187 ± 0.003^**^	0.199 ± 0.004^**^	–	–

“–” indicates that either no insects remained alive or that the number of alive individuals was insufficient for measurement.

Each insecticidal protein treatments was statistically compared with the control at each sampling date. Asterisks indicate significant differences between the toxin treatment and the control (^*^P < 0.05, ^**^P < 0.01).

**Table 2:** Body length and head width of *Folsomia candida* when fed artificial diets containing Cry1C, Cry2A, or E-64 protein for 7, 14, 21 or 28 days. Values are means ± SE, n = 40.

Treatment	Body length (mm)				Head width (mm)			
	7 day	14 day	21 day	28 day	7 day	14 day	21 day	28 day
Control: pure diet	0.906 ± 0.012	1.124 ± 0.019	1.300 ± 0.015	1.410±0.014	0.226 ± 0.002	0.243 ± 0.003	0.255 ± 0.003	0.275 ± 0.004
Cry1C 300 μg/g diet	0.883 ± 0.014	1.100 ± 0.016	1.282 ± 0.015	1.394±0.017	0.217 ± 0.003	0.239 ± 0.003	0.258 ± 0.004	0.270 ± 0.004
Cry2A 600 μg/g diet	0.870 ± 0.011	1.120 ± 0.013	1.258 ± 0.016	1.371±0.019	0.220 ± 0.003	0.237 ± 0.002	0.256 ± 0.002	0.273 ± 0.003
E-64 75 μg/g diet	0.849 ± 0.015^*^	1.110 ± 0.014	1.195 ± 0.012^**^	−	0.207 ± 0.003^**^	0.225 ± 0.003^**^	0.241 ± 0.003^*^	−

Statistical comparisons were made for each of the insecticidal protein with the control. Asterisks indicate significant differences between the toxin treatment and the control (^*^P < 0.05, ^**^P < 0.01).
