# Supplementary material for: Toxicological and biochemical analyses demonstrate no toxic effect of Cry1C and Cry2A to Folsomia candida
Source: Sci Rep. 2015 Oct 23;5:15619. doi: 10.1038/srep15619 (PMC4616050; doi:10.1038/srep15619)
Supplement: Supplementary Information [file srep15619-s1.doc]

**Supplementary Information**

Toxicological and biochemical analyses demonstrate that Cry1C and Cry2A are not toxic to *Folsomia candida*

Yan Yang, Xiuping Chen, Lisheng Cheng, Fengqin Cao, Jörg Romeis，Yunhe Li^**^, Yufa Peng

^1^State Key Laboratory for Biology of Plant Diseases and Insect Pests, Institute of Plant Protection, Chinese Academy of Agricultural Sciences, Beijing 100193, China

^2^College of Environment and Plant Protection, Hainan University, Haikou 570228, China

^3^Qiongtai Teachers College, Haikou 571127, China

^4^Agroscope, Institute for Sustainability Sciences ISS, 8046 Zurich, Switzerland

^*^ These authors contributed equally to this work.

^**^ Corresponding author: Yunhe Li

No. 2 West Yuanmingyuan Road, Haidian District, Beijing, China

E-mail: [yunhe.li@hotmail.com](mailto:yunhe.li@hotmail.com);

Tel: +86-10-62815947; Fax: +86-10-62896114

**Methods for bioassays with *C. suppressalis* larvae**

*Bt*-susceptible *C. suppressalis* larvae were used as sensitive insects to verify the bioactivity of the Cry1C and Cry2A proteins. Stock solution of Cry1C and Cry2A were diluted with distilled water and incorporated into an artificial diet as described in Han et al. (2012) for *C. suppressalis* to obtain the following concentrations: 0, 3.125, 6.25, 12.5, 25, 50, and 100 ng/g fresh weight (FW) of diet for Cry1C; 0, 50, 250, 1250, 2500, and 5000 ng/g FW of diet for Cry2A. These concentrations were selected based on our preliminary experiments. Since the *C. suppressalis* diet must be heated during preparation. To avoid the degradation of the Cry proteins during heating, the Cry protein solutions were mixed into the diet when the temperature had decreased to less than 60°C. Once the diet was solid, it was cut into slices and individually placed in Petri dishes (90 mm diameter, 15 mm height). Neonates of *C. suppressalis* were individually transferred to the Petri dishes, which were subsequently sealed with Parafilm. Thirty replicates were tested for each dose treatment. After 7 days, the *C. suppressalis* larvae were weighed using an electronic balance (CPA224S, Sartorius, Germany; d = 0.1 mg, ±0.1 mg). The EC50 (toxin concentration resulting in 50% weight reduction compared to the control) was estimated by probit analysis using the software package SPSS (version 13; SPSS, Inc., Chicago, IL).

**References**

Han, L.Z., Li, S.B., Liu, P.L., Peng, Y.F., Hou, M.L. New artificial diet for continuous rearing of *Chilo suppressalis* (Lepidoptera: Crambidae). *Ann. Entomol. Soc. Am.* **105**, 253–258 (2012).
